# Supplementary material for: Intestinal Microbiota and Derived Metabolites in Myocardial Fibrosis and Postoperative Atrial Fibrillation
Source: Int J Mol Sci. 2024 May 30;25(11):6037. doi: 10.3390/ijms25116037 (PMC11173100; doi:10.3390/ijms25116037)
Supplement: Supplementary file 1 [file ijms-25-06037-s001.zip › ijms-3015714-supplementary.pdf]

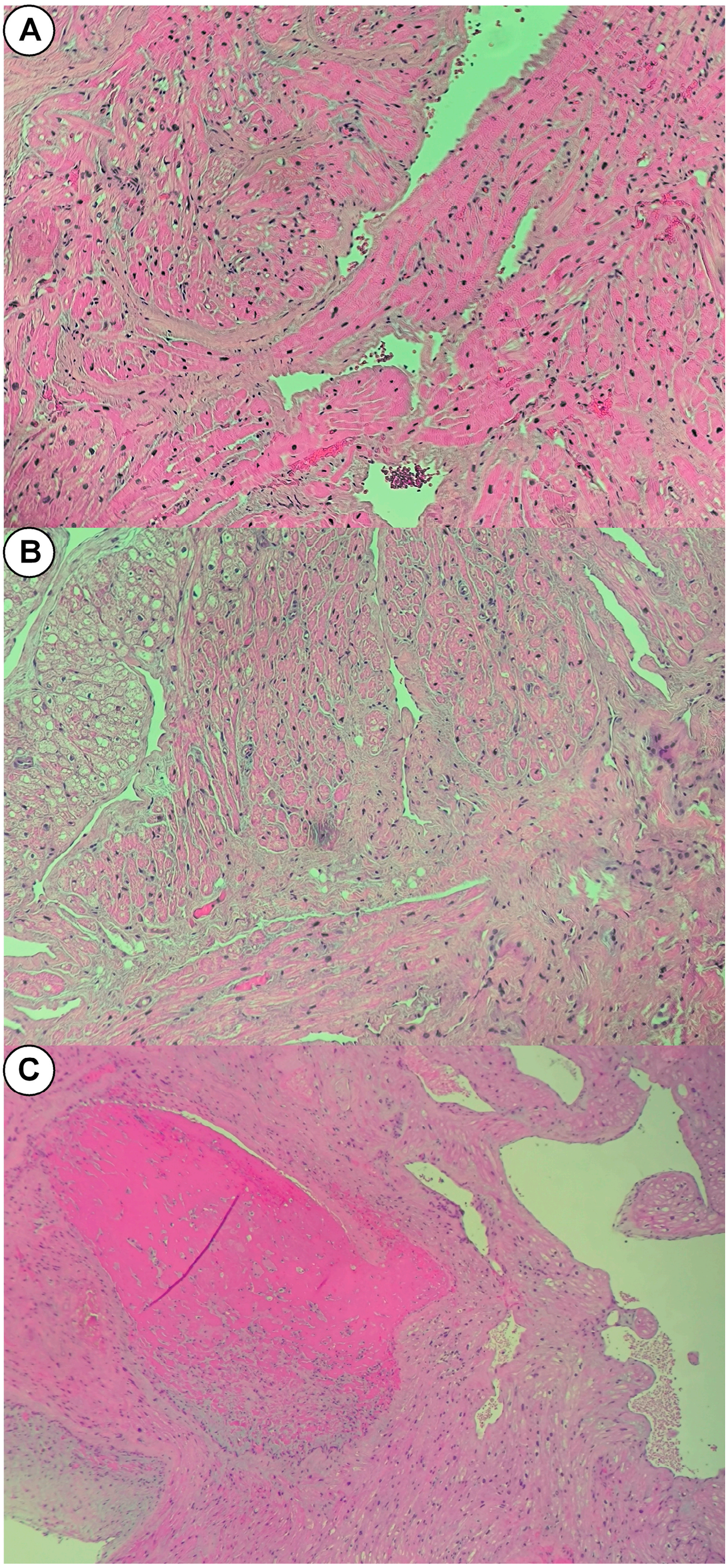

Supplementary Figure S1. Histologic evaluation of fibrosis of the right atrium (H/E, 10x): mild (A), moderate (B), severe (C).

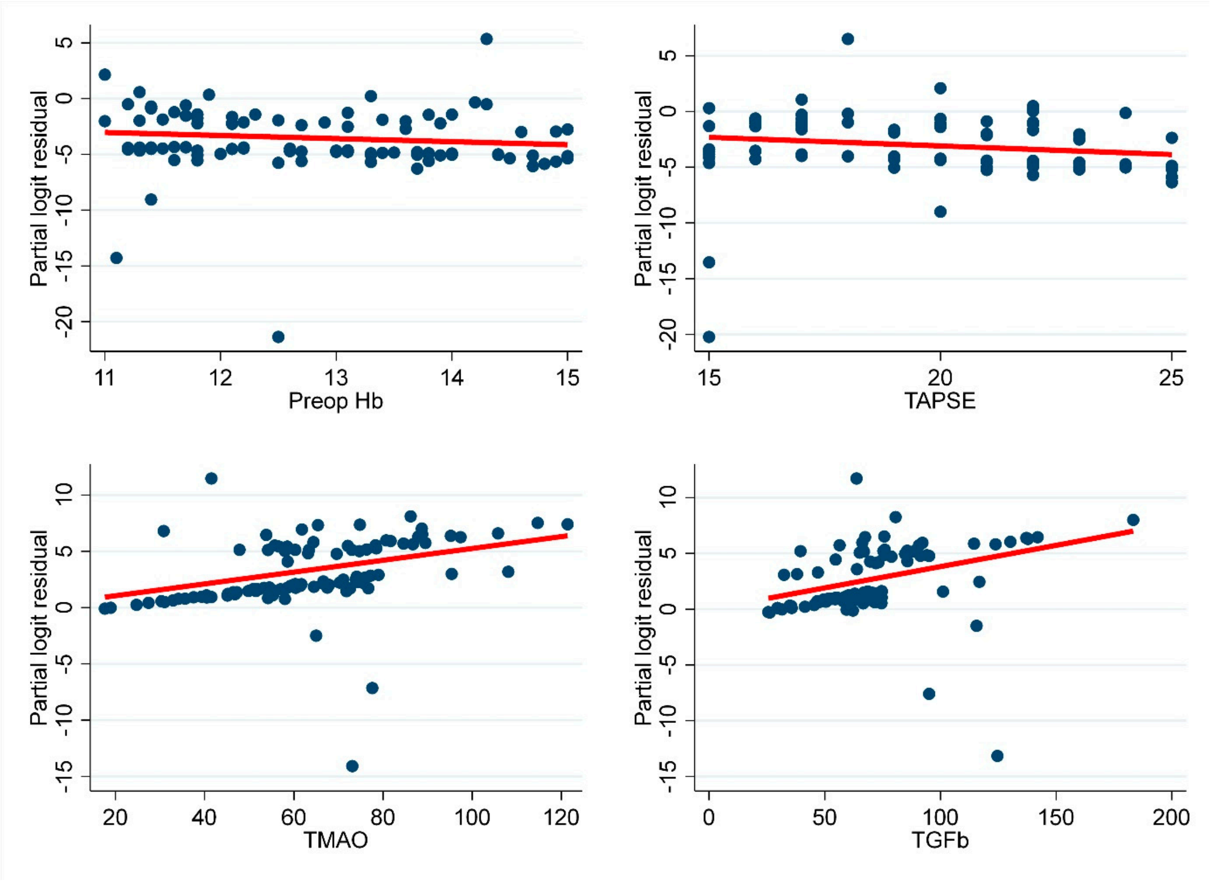

Supplementary Figure S2. Partial residual plot.

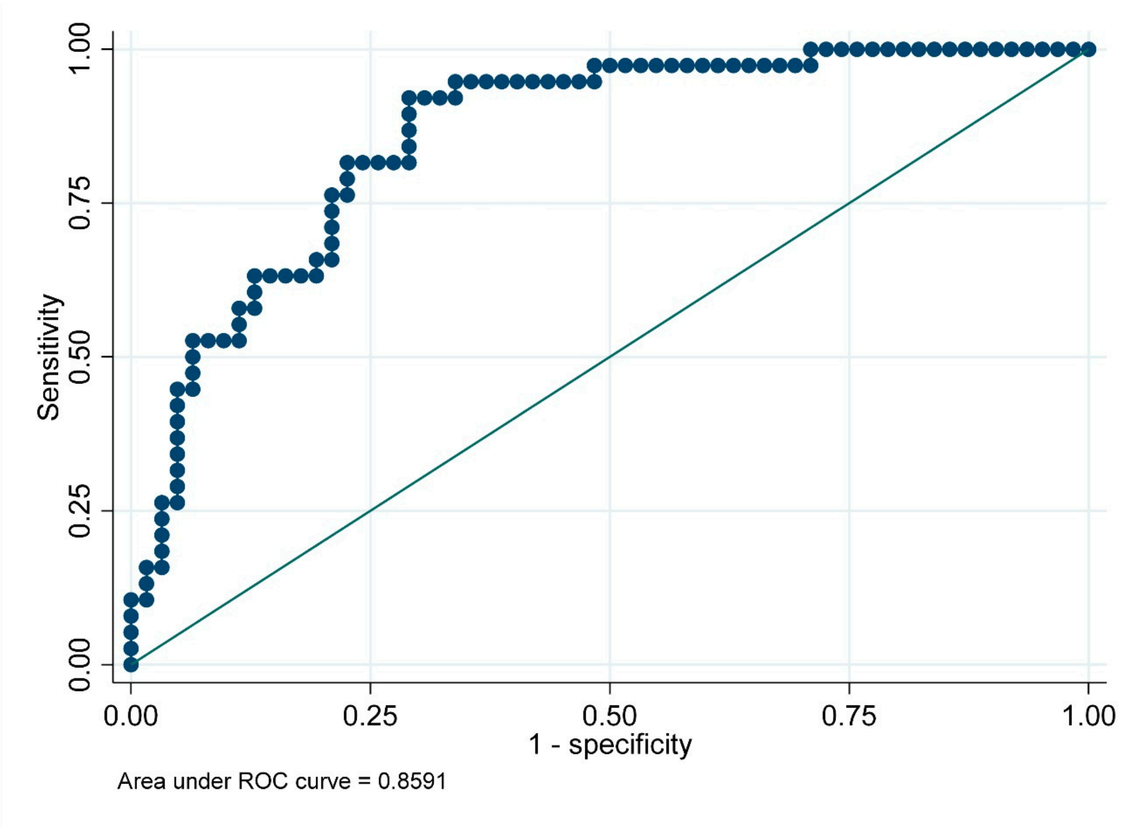

Supplementary Figure S3. ROC curve for the regression model in Table 6.

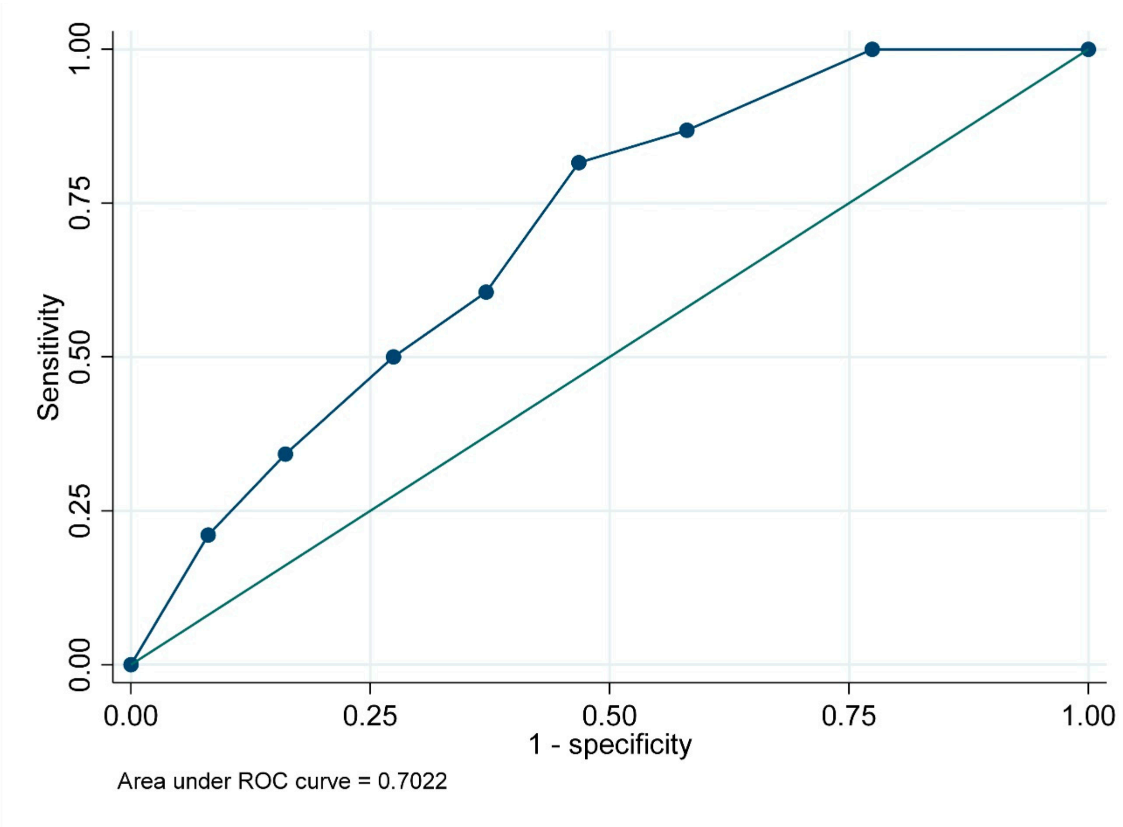

Supplementary Figure S4. ROC curve for the regression model in Table 9.
